# The Role of the Antidepressants in Managing Chemotherapy‐Induced Neuropathic Pain: A Systematic Review

**DOI:** 10.1002/prp2.70327

**Published:** 2026-09-17

**Authors:** Rosamaria Caminiti, Valeria Mazza, Saverio Nucera, Francesca Oppedisano, Lucia Carmela Passacatini, Jessica Maiuolo, Valentina Malafoglia, Alessandro Giorgio, Andrea Soluri, Claudia Pileggi, Carlo Tomino, Vincenzo Mollace, Sara Ilari, Carolina Muscoli

**Affiliations:** ^1^ Department of Health Sciences Institute of Research for Food Safety and Health (IRC‐FSH), University “Magna Graecia” of Catanzaro Catanzaro Italy; ^2^ Department of Medical and Clinical Surgery University Magna Græcia of Catanzaro Catanzaro Italy; ^3^ Pain Physiology and Pharmacology, IRCCS San Raffaele Roma Rome Italy; ^4^ Department of Experimental and Clinical Medicine “Magna Graecia” University Catanzaro Italy; ^5^ Department of Human Sciences and Quality of Life Promotion San Raffaele University Rome Italy

**Keywords:** antidepressants, chemotherapy drugs, chemotherapy‐induced peripheral neuropathy (CIPN), duloxetine, neuropathic pain, pain assessment, systematic review

## Abstract

Chemotherapy‐induced peripheral neuropathy (CIPN) is a prevalent and debilitating complication of cancer treatment, characterized by neuropathic pain, sensory disturbances, and functional impairment that can substantially reduce quality of life. Neurotoxicity induced by chemotherapeutic agents affects peripheral nerves, dorsal root ganglia, and supporting structures, contributing to persistent pain and neurological dysfunction. This systematic review evaluated the efficacy and safety of antidepressants, particularly duloxetine and amitriptyline, in the management of CIPN‐related pain. Following PRISMA guidelines and registration in PROSPERO (CRD42024608551), a systematic search of PubMed, Scopus, and Web of Science was conducted for studies published between November 2019 and October 2025. This time frame was selected to capture contemporary evidence and recent developments in multimodal treatment strategies, while acknowledging that foundational studies establishing the efficacy of duloxetine predate this period. Clinical and observational studies involving adult patients with CIPN were included. Of 892 records identified, 11 studies met the eligibility criteria. Duloxetine demonstrated clinically meaningful reductions in neuropathic pain, as assessed by VAS, NRS, and CTCAE measures. Topical amitriptyline also showed promising analgesic effects, although the available evidence remains limited. Combination therapies involving duloxetine and agents such as tapentadol, pregabalin, mirogabalin, or amitriptyline were associated with additional reductions in pain intensity; however, current evidence remains insufficient to establish additive or synergistic effects. Overall, antidepressant therapy was generally well tolerated, with predominantly mild and manageable adverse events. Current evidence supports antidepressants, particularly duloxetine, associated with reductions in pain intensity in heterogeneous studies, whereas evidence for CIPN prevention remains inconclusive. These findings support their continued use in clinical practice and highlight the need for larger, well‐designed randomized controlled trials to optimize treatment strategies and further define the role of combination therapies in CIPN management.

## Graphical abstract

From symptom relief to multimodal care: duloxetine and amitriptyline may reduce established CIPN‐related pain and provide a therapeutic foundation for future combination strategies.

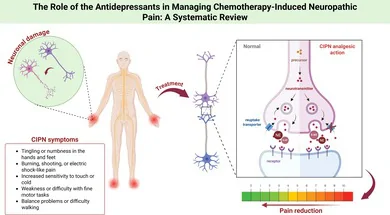

## 1 Introduction

Chemotherapy‐induced peripheral neuropathy (CIPN) is a form of neuropathic pain resulting from damage to the somatosensory nervous system caused by neurotoxic chemotherapeutic agents. According to the International Association for the Study of Pain (IASP), neuropathic pain is defined as “an unpleasant sensory and emotional experience associated with, or resembling that associated with, actual or potential tissue damage” [1]. The estimated global prevalence of CIPN is approximately 34% at 6 months or longer after chemotherapy. However, during the first month following treatment, prevalence may be as high as 68%, declining to approximately 60% at 3 months [2, 3, 4]. This condition is frequently difficult to manage and is associated with substantial impairment in quality of life, functional limitations, and significant social and economic consequences for affected patients.

CIPN may develop as a direct consequence of cancer itself or, more commonly, as an adverse effect of anticancer pharmacological treatments [5, 6]. Neurotoxicity is a well‐recognized adverse event of several widely used chemotherapeutic agents, including platinum‐based compounds, taxanes, bortezomib, thalidomide, and vinca alkaloids [7]. Neuropathic symptoms often persist or worsen after treatment discontinuation, a phenomenon referred to as “coasting” and may also emerge years after chemotherapy due to prolonged drug retention. Additional risk factors include alcohol abuse, genetic predisposition, environmental exposure, and lifestyle‐related factors [8, 9].

**FIGURE 1:**
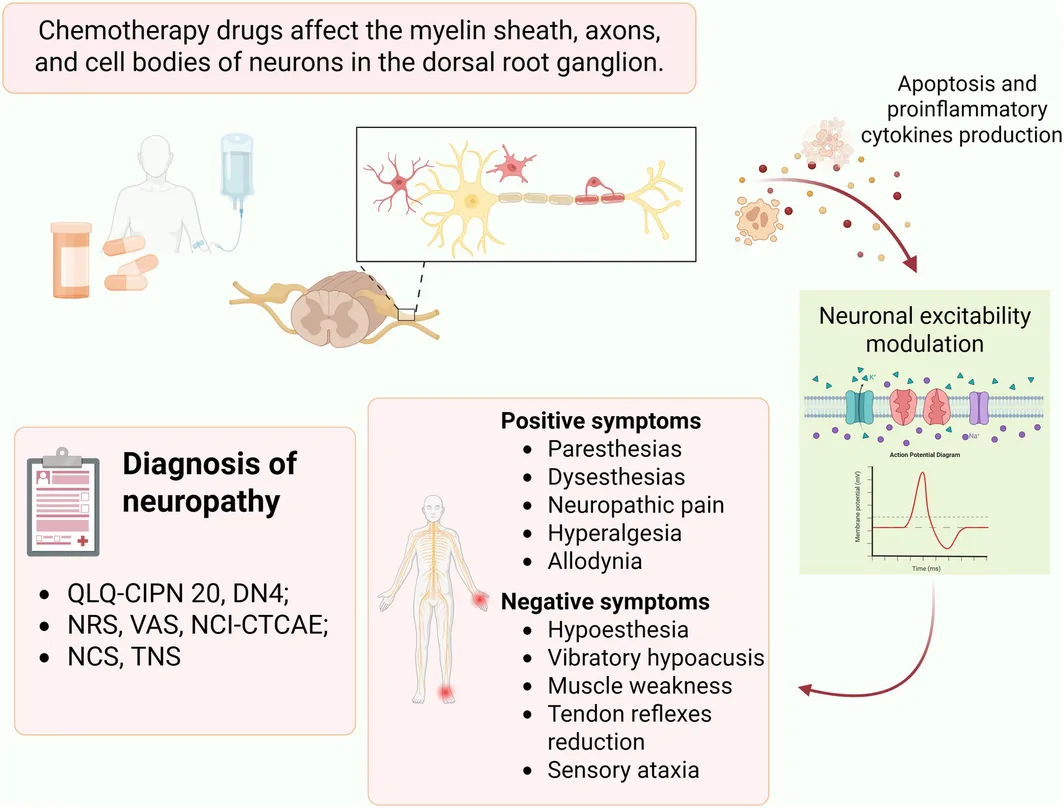
The figure outlines the pathophysiological mechanisms, clinical manifestations (positive and negative symptoms), and diagnostic approaches related to CIPN (Created in https://BioRender.com).

The pathophysiology of CIPN involves damage to the myelin sheath, axons, and neuronal cell bodies within the dorsal root ganglia (DRG), triggering neuroinflammation, increased production of pro‐inflammatory cytokines, apoptosis, and altered neuronal excitability [10]. Peripheral nerves are particularly vulnerable, explaining why symptoms predominantly affect the upper and lower extremities. Clinically, CIPN is characterized by both positive and negative sensory symptoms. Positive symptoms include allodynia, hyperalgesia, dysesthesia, and paresthesia, while negative symptoms include numbness and motor dysfunction (Figure 1).

Diagnosis relies on clinical history, patient‐reported outcome measures, and standardized tools. Common instruments include the EORTC Quality of Life Questionnaire–CIPN 20 (QLQ‐CIPN20), the Neuropathic Pain in 4 Questions (DN4), and pain scales such as the Numerical Rating Scale (NRS) and Visual Analogue Scale (VAS) [11]. Structured grading systems, including the National Cancer Institute Common Terminology Criteria for Adverse Events (NCI‐CTCAE) and the Total Neuropathy Score (TNS), integrate clinical and electrophysiological parameters to distinguish axonal from demyelinating neuropathies [7, 11].

Given the limited number of effective therapies, persistent symptoms in a substantial proportion of patients, and the increasing use of neurotoxic chemotherapeutic agents, there is a pressing need to define evidence‐based pharmacological strategies for CIPN management. Antidepressants, particularly those modulating serotonergic and noradrenergic pathways, have emerged as promising candidates due to their dual effects on pain modulation and mood regulation (Table 1). Clinical evidence remains heterogeneous, and the comparative efficacy and limitations of different antidepressant classes in CIPN are not fully clarified. Current international guidelines, ASCO and ESMO, report the absence of validated preventive interventions and identify duloxetine as the pharmacological treatment supported by the strongest evidence for painful CIPN, although its use remains off‐label [8, 12]. The efficacy of duloxetine in CIPN was first demonstrated in a randomized placebo‐controlled clinical trial conducted by Smith et al., which showed a significant reduction in pain scores compared to placebo [13]. This finding was later confirmed by Hirayama et al. in a large‐scale open‐label study [14].

**TABLE 1:** Antidepressants for CIPN management.

Drug	Class	Main effect in pain	Additional notes
Amitriptyline, Nortriptyline, Desipramine	TCAs	Analgesic effects by non‐selectively inhibiting the reuptake of 5‐HT and NE.	High risks of side effects such as dry mouth, weight gain, drowsiness, etc. Their use is limited [38]
Duloxetine	SNRI	Pain relief, inhibition of p38, NF‐kB, NGF. FDA‐approved for painful diabetic peripheral neuropathy [39]; its use in CIPN is off‐label but is supported by ASCO and ESMO guideline recommendations based on current clinical evidence [8].	Side effects include nausea, dizziness, somnolence, fatigue, hyperhidrosis, dry mouth, constipation, and diarrhea [39]
Venlafaxine	SNRI	Modulation of 5‐HT and NE transporters leads to relief of CIPN.	Low‐dose treatment has lower efficacy as it acts mainly as an SSRI. Recommended dose is roughly 75 mg two times a day. Variable results [40]

Abbreviations: 5‐HT, Serotonin; NE, Norepinephrine; SNRI, Selective serotonin‐noradrenaline reuptake inhibitor; SSRI, Selective Serotonin Reuptake Inhibitors; TCAs, Tricyclic antidepressants.

The 2019–2025 time window adopted in this review was selected for methodological reasons. First, it follows the publication of the updated guidelines, which serve as important reference points for contemporary clinical management of CIPN. Second, this period coincides with a growing focus on multimodal and combination‐based therapeutic approaches, which constitute the primary focus of the present review. Third, restricting the analysis to recent studies allows a more structured synthesis of evidence generated using relatively homogeneous diagnostic criteria and outcome reporting standards. It is acknowledged that the key evidence supporting the efficacy of duloxetine for CIPN originates from earlier studies. These studies are discussed to provide context and background but were not included in the primary evidence synthesis.

This work aims to critically synthesize and update the clinical evidence on the use of antidepressants, both selective and non‐selective, in managing CIPN. By integrating mechanistic insights with clinical outcomes, this work seeks to clarify therapeutic potential, highlight limitations, and identify gaps for future research and clinical decision‐making.

## 2 Materials and Methods

### 2.1 Database Sources

This systematic review aimed to evaluate the efficacy of antidepressant therapy in the management of chemotherapy‐induced neuropathic pain. Two investigators (Rosamaria Caminiti and Valeria Mazza) independently conducted searches on PubMed, Web of Science, and Scopus, following the PRISMA (Preferred Reporting Items for Systematic Reviews and Meta‐Analyses) guidelines and the PICO (Population, Intervention, Comparison, Outcome) framework.

The search strategy combined controlled vocabulary (MeSH terms in PubMed) and free‐text terms (synonyms, spelling variants, truncations) using Boolean operators (AND, OR).

- PubMed:
  (“neuropathic pain”[mh] OR “neuropathic pain”[tiab] OR neuropathy[tiab]) AND (“chemotherapy‐induced”[tiab] OR “chemotherapy‐related”[tiab] OR “antineoplastic agents/adverse effects”[mh] OR “chemotherapeutic agents”[tiab]) AND (antidepressant[tiab] OR “antidepressants”[mh] OR amitriptyline[tiab] OR duloxetine[tiab] OR nortriptyline[tiab] OR venlafaxine[tiab] OR “Serotonin and Norepinephrine Reuptake Inhibitors”[mh] OR “Tricyclic Antidepressants”[mh]) AND (“therapy”[sh] OR treatment[tiab] OR management[tiab] OR therapeutic use[mh:noexp])
- Scopus:
  (TITLE‐ABS‐KEY (“neuropathic pain” OR neuropathy)) AND (TITLE‐ABS‐KEY(“chemotherapy‐induced” OR “chemotherapy‐related” OR “chemotherapeutic agents”)) AND (TITLE‐ABS‐KEY(antidepressant OR amitriptyline OR duloxetine OR nortriptyline OR venlafaxine OR “serotonin norepinephrine reuptake inhibitors” OR “tricyclic antidepressants”)) AND (TITLE‐ABS‐KEY(treatment OR management OR therapy))
- Web of Science:
  (TS=(“neuropathic pain” OR neuropathy)) AND (TS=(“chemotherapy‐induced” OR “chemotherapy‐related” OR “chemotherapeutic agents”)) AND (TS=(antidepressant OR amitriptyline OR duloxetine OR nortriptyline OR venlafaxine OR “serotonin norepinephrine reuptake inhibitors” OR “tricyclic antidepressants”)) AND (TS=(treatment OR management OR therapy))

The review was registered on the PROSPERO platform (CRD42024608551). Initially, all articles were considered without restrictions to capture a comprehensive dataset. Subsequently, to focus on relevant evidence, only English‐language clinical studies published between 2019 and 2025 that were freely accessible were included. The last search was performed on 22 October 2025.

Additionally, the reference lists of all retrieved articles were screened to identify potentially eligible studies not indexed in the databases; no additional studies were found. Two independent reviewers (Rosamaria Caminiti and Valeria Mazza) screened all records against predefined inclusion and exclusion criteria. Reviewers were blinded to each other's decisions to reduce bias. Disagreements were resolved through discussion, and a third reviewer (Saverio Nucera) was consulted when consensus could not be reached.

A semi‐automated screening process was conducted using MySLR (version 1.0; https://myslr.unical.it, accessed 22 October 2025). Duplicate records were automatically identified and removed. Automated filtering was applied to exclude non‐original publications (reviews, editorials, books, and letters). The final eligibility assessment was performed manually by two independent reviewers, with full human verification of inclusion decisions. The platform supported organizational tasks such as record management and deduplication, while study selection remained entirely human‐driven [15, 16, 17].

### 2.2 Eligibility Criteria

Included studies were clinical investigations: randomized controlled trials (RCTs), observational studies (case–control, cohort, cross‐sectional), longitudinal studies, including follow‐up and retrospective analyses. Eligible patients were ≥ 18 years old. Excluded were in vitro/in vivo studies, narrative reviews, books, chapters, editorials, meta‐analyses, and systematic reviews. Studies were also excluded if:

- They involved cancer patients receiving pain therapies without antidepressants for CIPN management;
- Pain was not chemotherapy‐induced;
- Antidepressant therapy was unrelated to CIPN management (Table 2).

**TABLE 2:** Study inclusion and exclusion criteria.

Category	Inclusion criteria	Exclusion criteria
Population	Adult patients (over 18 years of age) Cancer patients with CIPN	Pediatric patients (18 years or younger)
Intervention	Pain management involving antidepressants Other pharmacological or non‐pharmacological interventions for CIPN included if compared to antidepressants	Pain not caused by chemotherapy Antidepressant therapy unrelated to CIPN management
Comparison	Placebo Other drugs (e.g., pregabalin, tapentadol) Sham interventions (e.g., sham electrostimulation)	Studies without a comparator or non‐relevant comparator
Outcome	Pain reduction (assessed by NRS, VAS) CIPN improvement (assessed by CTCAE, DN4)	Studies not reporting pain or CIPN outcomes
Study design	Clinical studies, including: RCTs Observational studies (case–control, cohort, cross‐sectional, longitudinal) Follow‐up and retrospective studies	In vitro and in vivo studies Narrative reviews, books, chapters, editorials Meta‐analyses and systematic reviews

Abbreviations: CTCAE, Common Terminology Criteria for Adverse Events (v4.03 or v5.0); DN4, Douleur Neuropathique en 4 Questions; NRS, Numeric Rating Scale; RCTs, Randomized Controlled Trials; VAS, Visual Analogue Scale (scale 0–10).

### 2.3 Study Outcomes

The primary objective was to assess the analgesic efficacy of antidepressants in cancer patients with chemotherapy‐induced neuropathic pain. Secondary objectives included evaluation of improvement in overall disability and quality of life. Pain intensity was measured using the Numerical Rating Scale (NRS), Visual Analogue Scale (VAS), and CIPN grading according to CTCAE criteria. Based on IMMPACT criteria, a clinically significant difference was defined as a reduction of at least 2 points on an 11‐point NRS scale or a reduction of at least 30% in pain score from baseline [18].

### 2.4 Data Synthesis

Due to heterogeneity in study designs, interventions, and outcome measures, a quantitative meta‐analysis was not feasible. Findings were synthesized qualitatively and stratified by study design and type of intervention.

### 2.5 Data Extraction and Qualitative Synthesis

The synthesis process was structured as follows.

#### 2.5.1 Data Extraction

Two independent reviewers (Rosamaria Caminiti and Valeria Mazza) extracted data using a standardized form. The extracted data included: study design, sample characteristics including tumor type and sample size; intervention details such as drug, dosage, duration, comparator, primary and secondary outcomes, and reported adverse events.

#### 2.5.2 Thematic Grouping

The extracted data were organized to facilitate comparison and integration by type of intervention. Studies were grouped into duloxetine monotherapy, other antidepressant monotherapies (e.g., amitriptyline), combination therapies (e.g., duloxetine + pregabalin/mirogabalin/tapentadol/other antidepressants), topical treatments (e.g., 10% amitriptyline gel). Furthermore, studies were divided by outcome domain: within each intervention group, results were summarized by efficacy on pain, changes in pain intensity scales (NRS, VAS) and by efficacy on neuropathy severity through changes in CIPN‐specific scores (CTCAE, TNS, QLQ‐CIPN20). Safety and tolerability were briefly described with type and frequency of adverse events.

#### 2.5.3 Narrative Integration

Within each thematic group, results were compared and contrasted. The consistency and magnitude of effects across studies were noted. Discrepancies or divergent results were explicitly highlighted and contextualized, considering differences in study design, population, dosage, or treatment duration. This narrative approach allowed us to identify patterns (e.g., consistent benefit of duloxetine, additive effect of combinations) and evidence gaps, forming the basis for a structured discussion.

### 2.6 Risk of Bias Assessment

The risk of bias in the included studies was assessed using the Cochrane RoB 2 tool for randomized controlled trials (RCTs) and the Newcastle‐Ottawa Scale for observational studies. Most RCTs demonstrated a low risk of bias across key domains, including randomization, blinding of participants and outcome assessors, and outcome measurement. Some studies, however, presented “some concerns” regarding missing data or deviations from intended interventions, whereas observational studies and case reports inherently carried a higher risk of bias (Table 3).

**TABLE 3:** Quality assessment.

Author (year)	Study design	Randomization/selection	Deviations from intended treatment	Missing outcome data	Outcome measurement	Reported outcomes	Overall judgment
Aghili (2024)	Double‐blind RCT	Low risk	Low risk	Some concerns	Low risk	Low risk	Low/Some concerns
Rokhsareh (2023)	Double‐blind RCT	Low risk	Low risk	Some concerns	Low risk	Low risk	Low/Some concerns
Matsuoka (2019)	Multicenter, double‐blind RCT	Low risk	Low risk	Some concerns	Low risk	Low risk	Low/Some concerns
Kuroyanagi (2024)	Case report	N.A.	N.A.	N.A.	N.A.	N.A.	High risk
Song (2020)	Randomized study, ES device + drugs	Low risk	Low risk	Some concerns	Low risk	Low risk	Some concerns
Singhal (2025)	Prospective RCT	Low risk	Some concerns	Some concerns	Low risk	Low risk	Low/Some concerns
Saito (2022)	Case report	N.A.	N.A.	N.A.	N.A.	N.A.	High risk
Nakajima (2025)	Retrospective observational study	Some concerns (Newcastle–Ottawa)	Some concerns	Some concerns	Some concerns	Low risk	Some concerns
Coffen (2019)	Retrospective observational study	Some concerns (Newcastle–Ottawa)	Some concerns	Some concerns	Some concerns	Low risk	Some concerns
Sansone (2022)	Double‐blind RCT	Low risk	Low risk	Some concerns	Low risk	Low risk	Low/Some concerns
Rossignol (2019)	Pilot study/topical antidepressant TC	Some concerns	Some concerns	Some concerns	Low risk	Low risk	Some concerns

Abbreviations: ES, experimental electrostimulation setting; N.A., not available; RCT, randomized controlled trial.

Despite these concerns, the overall methodological quality of the RCTs provides robust evidence supporting the efficacy of duloxetine and amitriptyline in the management of CIPN. The inclusion of studies with varying levels of bias was carefully considered in interpreting the findings, ensuring a balanced and transparent representation of the current literature.

## 3 Results

### 3.1 Study Selection and Characteristics

**FIGURE 2:**
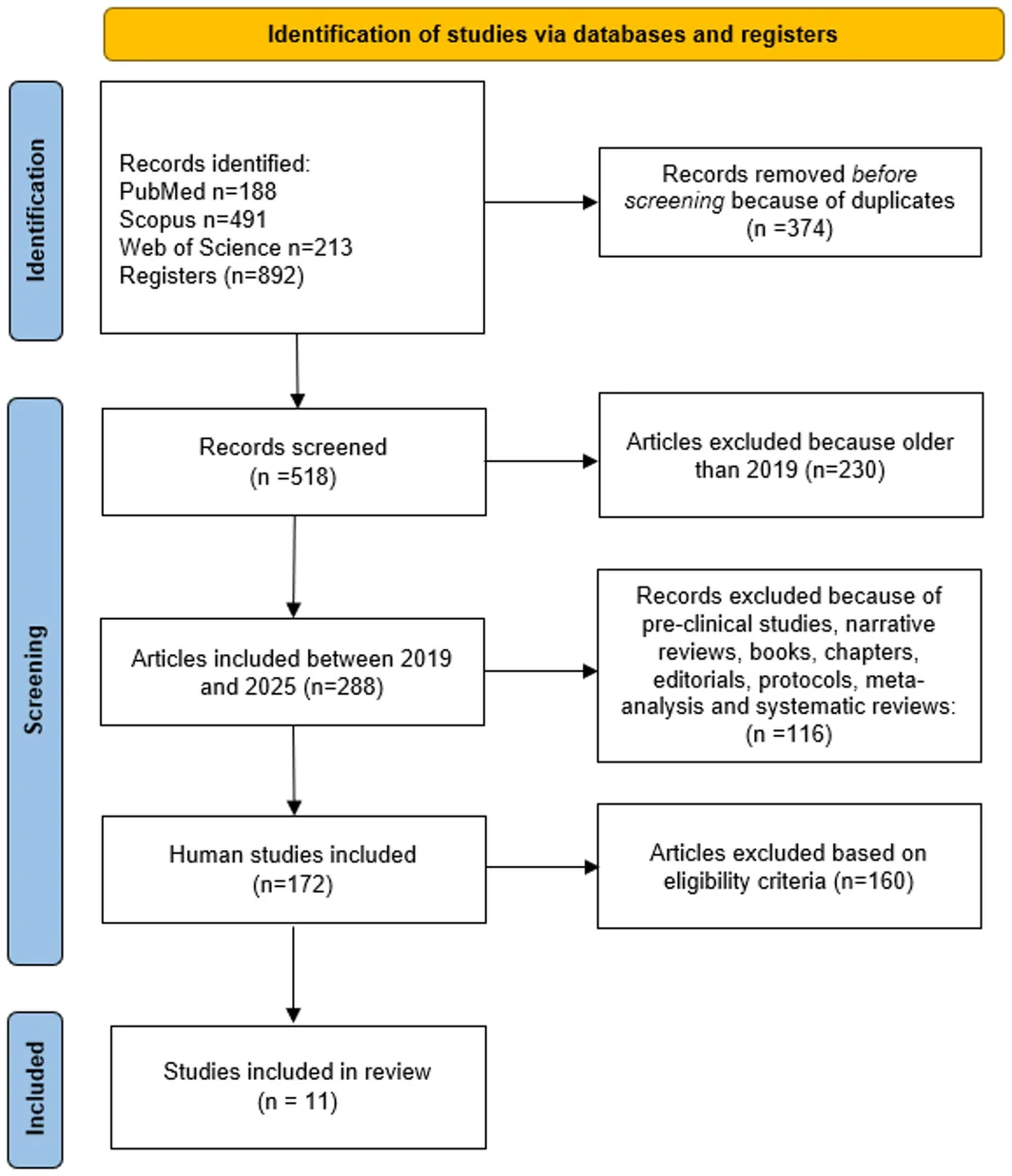
PRISMA 2020 flow diagram for new systematic reviews, which included searches of databases and registers only.

A total of 892 records were identified through database searching. After removal of 374 duplicates, 518 records remained. Following title and abstract screening, 230 studies published before 2019 were excluded, and 116 additional records were excluded for being in vitro or in vivo studies, reviews, books, chapters, editorials, meta‐analyses, or systematic reviews. Of the remaining 172 articles, those not freely accessible or not relevant to eligibility criteria were excluded, leaving 11 clinical studies for qualitative synthesis (Figure 2).

The included studies comprised (Table 4):

- 3 randomized, double‐blind, placebo‐controlled trials
- 1 randomized controlled trial
- 1 randomized, non‐inferiority clinical trial
- 2 case reports
- 1 prospective pilot study
- 1 prospective randomized controlled trial
- 1 retrospective file‐based observational study
- 1 retrospective observational cohort study

**TABLE 4:** Main characteristics of the studies included in the review.

Enrolled patients	Patients at the end of the study	Average age	Tumor	Type of study	Study duration	References
47 (24 placebo, 23 drug)	47	45.89	Non‐metastatic breast cancer	Randomized double‐blind placebo controlled clinical trial	September 2019 – September 2021	[19]
64 (31 placebo, 33 drug)	40 (20 placebo, 20 drug)	59.6 placebo, 55.75 drug	Gastrointestinal cancer	Randomized double‐blind placebo controlled clinical trial	July 2016–August 2018	[20]
70	65 (29 placebo, 32 duloxetine)	62.9 ± 10.6	Head and neck, thoracic, gastrointestinal, genitourinary cancer	Randomized controlled trial	N.A.	[21]
1	1	49	Breast cancer	Case report	N.A.	[28]
72 (36 ES, 36 SES)	64 (30 ES, 34 SES)	50 ± 8.85	Breast cancer	Randomized placebo‐controlled trial	14 days	[23]
1	1	53	Breast cancer	Case report	N.A.	[24]
754	233	58 ± 12.4	Breast, cervix, hematological, lung and colon cancers	Retrospective file‐based observational study	January 2016–January 2017	[22]
114 (56 TP, 58 TP + DLX)	108 (52 TP, 56 TP + DLX)	52.4 TP, 51.7 TP + DLX	Solid (37 breast, 27 digestive, 12 respiratory system, 11 gynecological, 9 oropharynx); non‐solid,12	Randomized non‐inferiority clinical trial	May 2018–November 2019	[25]
44	22	67 ± 8.5	Hematological, 39; solid 5	Prospective, pilot study	12 months	[29]
100	89 (45 D, 44 P)	D: 49.3 ± 5.2; P: 51.6 ± 9.4	Lung, breast, and oral cancer	Prospective randomized controlled trial	4 weeks	[26]
14	14	71 ± 9.3	Lung cancer	Retrospective observational cohort study	Registration: April 2020–July 2022; data analysis period: June 2023–December 2023	[27]

Abbreviations: DLX, duloxetine; ES, Experimental electrostimulation Setting; N.A., Not Available; P, Pregabalin; SES, Sham electrostimulation Setting; TP, tapentadol.

Study populations included patients with breast, gastrointestinal, lung, hematological, and other solid and non‐solid tumors. Sample sizes ranged from 1 (case reports) to 231 participants.

### 3.2 Antidepressant Interventions

Antidepressants evaluated included duloxetine, amitriptyline, venlafaxine, and combination therapies with mirogabalin or tapentadol. Dosages ranged as follows (Table 5):

- Duloxetine: 20–60 mg per day.
- Amitriptyline: topical 10%, and os 10–20 mg per day.
- Tapentadol: 50–500 mg per day (alone or with duloxetine).
- Mirogabalin: 5–25 mg per day (co‐administered with duloxetine).

**TABLE 5:** Therapy sessions conducted by cancer patients in the studies included.

Chemotherapy therapy	Antidepressant (or combinations)	Dose	Treatment duration	References
Adriamycin + cyclophosphamide and paclitaxel	Duloxetine	30 mg per day (1 week after chemotherapy), 60 mg (2 weeks after chemotherapy)	8 weeks	[19]
Oxaliplatin	Duloxetine	30 mg per day (1 week); 60 mg per day (up to 12 weeks)	12 weeks	[20]
N.A.	Duloxetine	20 mg (up to the third day); then 40 mg	10 days	[21]
Paclitaxel	Sympathetic nerve block + duloxetine + amitriptyline	Duloxetine: 20 mg daily up to 60 mg/day; amitriptyline: 20 mg daily up to 10 mg/day (in association with nerve block)	40 months	[28]
Docetaxel + doxorubicin + cyclophosphamide	Pregabalin, duloxetine, or both	N.A.	N.A.	[23]
Eribulin, docetaxel and cyclophosphamide	Mirogabalin + duloxetine	Mirogabalin: 25 mg per day; duloxetine: 20 mg per day	N.A.	[24]
Platinum, cisplatin, vincristine, paclitaxel, docetaxel, thalidomide, lenalidomide	Duloxetine	N.A.	N.A.	[22]
Taxanes or platinum‐based	Tapentadol/duloxetine + tapentadol	Tapentadol: 50 to 500 mg per day; duloxetine+tapentadol: 60 to 120 mg per day	42 days	[25]
Bortezomib and oxaliplatin	Amitriptyline	1 g 10% topical amitriptyline, emulsion (or/a), 2 times daily	6 months	[29]
Paclitaxel alone or with carboplatin, cisplatin, vincristine, or bortezomib	Duloxetine + pregabalin	Duloxetine: 30 mg daily (first week) then 30 mg twice daily; pregabalin: 75 mg daily (first week), then 75 mg twice daily	4 weeks	[26]
Taxanes	Duloxetine + mirogabalin	Duloxetine (20 mg daily) and mirogabalin (5 mg twice daily, increased by 5 mg per week, maximum dose of 15 mg twice daily)	Duloxetine: for the entire observation period; mirogabalin (32 days, range 7–84)	[27]

Abbreviation: N.A., not available.

### 3.3 Clinical Outcomes

The results of the selected studies are presented in Table 6.

**TABLE 6:** Changes in CIPN symptoms were assessed through evaluation scales after antidepressant treatment.

Pain assesment scale baseline	Pain assessment scale after treatment	CIPN baseline	CIPN after treatment	References
NRS: 4.19	2.63	CTCAE v5.0: 1.48	0.79	[19]
N.A.	N.A.	CTCAE v4.03: 0.86	0.62 (*p* = 0.025)	[20]
BPI‐Item 5: 5.6 ± 1.4	−1.30 (CI 95%: −1.98 to −0.63)	N.A.	N.A.	[21]
NRS: 8	3	CTCAE v5.0: 3	2	[28]
NRS: 6.1 ± 1.1(ES) 6.5 ± 1.4(SES)	3.6 ± 2.2 (ES) 4.6 ± 2.6 (SES)	N.A.	N.A.	[23]
NRS: 8	5 (MRG) 1 (MRG + DLX)	CTCAE v5.0: 3	1	[24]
VAS: 2.5 ± 2.6	2.3 ± 2.5 (*p* > 0.05)	N.A.	N.A.	[22]
NRS: 7.48 (TP) 7.51 (TP + DLX)	3.02 (−4.46; TP) 2.87 (−4.64; TP + DLX); (*p* = 0.93)	N.A.	N.A.	[25]
VAS: 7	2 (*p* < 0.0001)	N.A.	N.A.	[29]
NRS: 7.04 ± 0.903 (DLX); 6.98 ± 0.920 (P) (*p* = 0.416)	4.04 ± 0.99 (DLX); 4.91 ± 0.960 (P) (*p* < 0.001)	DN4: 7.02 ± 0.917 (DLX); 6.93 ± 0.85 (P) (*p* = 0,145)	3.87 ± 0.694 (DLX); 5.02 ± 1.023 (P) (*p* < 0.001)	[26]
NRS: 5.5 (4.5–7.0)	4.0 (3.0–5.0) (*p* = 0.041)	N.A.	N.A.	[27]

Abbreviations: BPI‐Item 5, Brief Pain Inventory; CTCAE, Common Terminology Criteria for Adverse Events (v4.03 or v5.0); DLX, duloxetine; DN4, Douleur Neuropathique en 4 Questions; ES, Experimental electrostimulation Setting; MRG, Mirogabalin; N.A., Not Available; NRS, Numeric Rating Scale; P, Pregabalin; SES, Sham electrostimulation Setting; TP, tapentadol; VAS, Visual Analogue Scale (scale 0–10).

#### 3.3.1 Duloxetine Monotherapy

Aghili et al.: NRS decreased from 4.19 to 2.63; CTCAE from 1.48 to 0.79 in 23 patients treated with duloxetine versus 24 placebo (*p* = 0.027) [19].

Rokhsareh et al.: in 40 gastrointestinal cancer patients, duloxetine reduced CIPN grade from 0.86 to 0.62; peripheral sensory neuropathy scores were lower compared to placebo (*p* = 0.01) [20].

Matsuoka et al.: clinically significant pain reduction (≥ 30%) in 44.1% of duloxetine‐treated patients versus 18.2% placebo; ≥ 50% reduction in 32.4% versus 3.0% (*p* = 0.002) [21].

Coffen et al.: VAS decreased from 2.5 ± 2.6 to 2.3 ± 2.5; reduction not statistically significant (*p* > 0.05) [22].

#### 3.3.2 Combination Therapy

Song et al.: Duloxetine plus electrostimulation reduced NRS from 6.1 ± 1.1 to 3.6 ± 2.2 (*n* = 72) [23].

Saito et al.: Mirogabalin plus duloxetine reduced NRS from 8 to 1 and CTCAE score from 3 to 1 (*n* = 1) [24].

Sansone et al.: Tapentadol combined with duloxetine reduced NRS from 7.51 to 2.87 (−4.64; *p* = 0.93) (*n* = 114) [25].

Singhal et al.: Duloxetine reduced NRS from 7.04 ± 0.903 to 4.04 ± 0.99 (*p* < 0.001); pregabalin from 6.89 ± 0.920 to 4.91 ± 0.960 (*p* < 0.001) (*n* = 89) [26].

Nakajima et al.: Mirogabalin plus low‐dose duloxetine reduced NRS from 5.5 (IQR 4.5–7.0) to 4.0 (IQR 3.0–5.0; *p* = 0.041) [27].

Kuroyanagi et al. in a case report of a patient with paclitaxel‐induced CIPN, described the effect of sympathetic nerve block in combination with duloxetine and oral amitriptyline. The NRS score was reduced from 8 to 3 and the CTCAE grade from 3 to 2. However, since nerve block represented the main intervention, it is not possible to attribute the observed benefit solely to antidepressants [28].

#### 3.3.3 Topical Amitriptyline

Rossignol et al.: 10% topical amitriptyline twice daily reduced VAS from 7 to 2 over 4 weeks (*n* = 44) [29].

#### 3.3.4 Adverse Events

- Duloxetine: nausea, constipation, dizziness, mild and generally not dose‐limiting.
- Combination therapies did not increase the incidence of adverse events relative to monotherapy.
- Tapentadol, pregabalin, and mirogabalin co‐administration were well tolerated with mild fatigue, nausea, or drowsiness.

### 3.4 Summary

Among the antidepressants evaluated, duloxetine was the most extensively studied agent, administered at daily doses ranging from 20 to 60 mg, followed by topical amitriptyline 10% and venlafaxine. These agents were used either as monotherapy or in combination with other analgesics, including mirogabalin or tapentadol. Combination regimens primarily consisted of duloxetine co‐administered with mirogabalin (5–20 mg daily) or tapentadol (50–500 mg daily).

Duloxetine monotherapy consistently demonstrated significant reductions in pain intensity and neuropathy severity compared with placebo, with adverse events generally mild and predominantly limited to nausea, constipation, and dizziness. Topical amitriptyline also exhibited promising analgesic efficacy in a prospective pilot study. Overall, combination therapies tended to yield greater pain relief without increasing the incidence of adverse events relative to monotherapy. Co‐administration with tapentadol, pregabalin, or mirogabalin was well tolerated, with only mild and transient episodes of fatigue, nausea, or somnolence.

## 4 Discussion

This systematic review explored the therapeutic role of antidepressants in managing CIPN, drawing on current evidence from the literature. The qualitative evidence summarized here should be interpreted in the context of earlier landmark studies, in particular the randomized trial by Smith et al., which first demonstrated duloxetine's efficacy in CIPN, and the study by Hirayama et al., which provided supportive evidence in an independent Japanese cohort [13, 14]. Preclinical studies provide mechanistic support for these clinical observations, as duloxetine, amitriptyline, and pregabalin reduce neuropathic pain behaviors in animal models. However, translational inconsistencies persist, particularly for pregabalin and venlafaxine, which show stronger effects in preclinical models than in clinical trials [30]. These discrepancies may reflect differences in treatment timing, dosing strategies, outcome measures, and the limited ability of current animal models to reproduce the complexity of human CIPN, underscoring the need for cautious interpretation when translating animal data to clinical CIPN management.

The studies included in this review addressed a condition that substantially impairs quality of life and may necessitate chemotherapy dose reduction or discontinuation. The aim was to evaluate the relationship between CIPN induced by agents such as taxanes, platinum compounds, and alkylating agents, and the potential role of antidepressants in symptom control. Overall, duloxetine demonstrated benefit, whereas amitriptyline showed potential efficacy but is supported by considerably less robust evidence.

From the screening conducted, eleven articles were identified that met the inclusion criteria, ensuring the use of the most recent and relevant data. Rather than focusing on the clinical presentation of CIPN, which is already well established, the present review sought to evaluate whether antidepressant therapy can meaningfully improve neuropathic symptoms and patient‐reported outcomes. Given the substantial burden of CIPN on daily functioning and quality of life, effective symptomatic treatment remains an important unmet clinical need [3, 29]. Current guidelines recommend duloxetine as the first‐line pharmacological treatment for CIPN, based on moderate‐certainty evidence and supported by a conditional recommendation [8, 12].

Across the included trials, duloxetine generally improved pain scores, although results varied with treatment duration and study design [19, 20, 21, 22]. Taken together, these studies suggest a favorable effect of duloxetine on CIPN‐related pain, although the magnitude of benefit appears to be influenced by methodological factors, treatment duration, and sample size. Aghili et al. reported reductions in NRS (4.19 to 2.63) and CTCAE scores (1.48 to 0.79) after 8 weeks [19]. Rokhsareh et al. observed prevention of symptom worsening compared with placebo in gastrointestinal cancer patients [20]. In contrast, Matsuoka and Coffen did not report statistically significant differences, likely due to short treatment duration and limited drug availability [21, 22].

Although several studies [23, 24, 25, 26, 27, 28] reported greater reductions in pain intensity when duloxetine was combined with agents such as tapentadol, pregabalin, mirogabalin, electrostimulation, or other supportive interventions, the available evidence does not allow conclusions regarding additive or synergistic effects. Most studies were not specifically designed to evaluate treatment interactions and typically investigated multimodal interventions without appropriate comparator arms. Consequently, it remains unclear whether the observed benefits reflect the independent contribution of each component or an interaction exceeding their expected individual effects. Therefore, any suggestion of additive or synergistic efficacy should be regarded as hypothesis‐generating rather than established.

Duloxetine shows consistent monotherapy efficacy [13, 19, 21], whereas mirogabalin lacks randomized placebo‐controlled evidence in CIPN, being limited to retrospective and observational data [24, 27]. Pregabalin shows strong preclinical efficacy but limited clinical effectiveness, suggesting a translational gap possibly related to dosing, duration, and patient selection. Pharmacodynamic differences, including receptor affinity and onset of action between pregabalin and mirogabalin, may partly explain preliminary differences, although evidence remains inconclusive.

The predominance of duloxetine among effective agents may be partially explained by its pharmacological mechanism of action. Mechanistically antidepressant analgesia in CIPN is thought to involve modulation of descending inhibitory pathways, particularly noradrenergic projections from the locus coeruleus to the spinal dorsal horn. Serotonin‐Norepinephrine Reuptake Inhibitors (SNRIs) (i.e., duloxetine) and Tricyclic Antidepressants (TCAs) (i.e., amitriptyline) enhance both serotonergic and noradrenergic transmission, whereas Selective Serotonin Reuptake Inhibitors (SSRIs) predominantly act on serotonergic pathways and lack substantial engagement of descending noradrenergic inhibitory circuits, which has been proposed as one potential explanation for their limited and inconsistent efficacy in neuropathic pain conditions, including CIPN. However, the available clinical evidence is heterogeneous, and class‐level effects cannot be conclusively established [31].

Although SSRIs appear to be ineffective as monotherapy in neuropathic pain, preclinical studies have suggested a potential synergistic effect when combined with opioids or other analgesic agents. As early as 1990, Porreca et al. hypothesized a synergistic interaction between opioids and SSRIs based on preclinical findings. Subsequent studies [31, 32] have further suggested convergence on descending inhibitory pathways and calcium channel modulation, supporting a possible adjuvant role of SSRIs. However, clinical evidence supporting such combinations, particularly in CIPN, remains lacking. Topical amitriptyline may provide localized analgesia with improved tolerability compared to systemic administration, though evidence remains limited to small studies and case reports [33, 34].

To contextualize the magnitude of the observed benefit, it is useful to compare the findings of the studies included in this review with those reported in other neuropathic pain conditions. Across the studies examined, duloxetine, administered either as monotherapy or in combination with other treatments, was associated with reductions in pain intensity. These findings are consistent with evidence from other neuropathic pain conditions, such as painful diabetic neuropathy and postherpetic neuralgia, in which systematic reviews have demonstrated a comparable magnitude of benefit with duloxetine treatment [35, 36].

## 5 Conclusions and Limitations of the Study

This systematic review has several methodological limitations that should be acknowledged. First, the included studies were highly heterogeneous with respect to study design, patient populations, concomitant treatments, and outcome measures, precluding quantitative synthesis and necessitating cautious interpretation of treatment effects. The predominance of small sample sizes and observational designs further limits the robustness and generalizability of the conclusions.

Second, the review was restricted to studies published between 2019 and 2025 in English and available through open‐access sources. Although this approach was intended to capture contemporary multimodal and combination‐treatment strategies, it may have introduced language and publication‐access bias and excluded foundational studies that established the evidence base for duloxetine in CIPN. Current evidence supports the use of antidepressants primarily for the treatment of established CIPN‐related pain, whereas their role in preventing CIPN remains unproven [37]. Despite these limitations, the available evidence identifies duloxetine as the current first‐line pharmacological option, although the overall quality of the evidence remains moderate and heterogeneous. Emerging data on combination therapies suggest that greater pain reduction may be achievable in some patients; however, the available evidence is insufficient to determine whether these effects are additive, synergistic, or primarily driven by one component of the treatment regimen. Dedicated randomized controlled trials are needed to clarify the contribution of individual interventions and the potential value of multimodal therapeutic approaches.

Future research should focus on large‐scale randomized controlled studies with longer follow‐up periods, standardized patient‐centered outcome measures, and rigorous evaluation of both monotherapy and combination‐therapy strategies. The development of effective preventive interventions, together with reliable diagnostic and monitoring tools, remains essential for improving long‐term clinical outcomes, quality of life, and functional recovery in patients with chemotherapy‐induced peripheral neuropathy.

## Author Contributions

**Rosamaria Caminiti:** conceptualization, investigation. **Valeria Mazza:** validation, investigation, conceptualization. **Saverio Nucera:** methodology, investigation. **Francesca Oppedisano:** conceptualization, visualization, writing – original draft. **Lucia Carmela Passacatini:** writing – review and editing, writing – original draft, conceptualization. **Jessica Maiuolo:** methodology, conceptualization, writing – review and editing, writing – original draft. **Valentina Malafoglia:** funding acquisition, methodology, conceptualization. **Alessandro Giorgio:** visualization. **Andrea Soluri:** methodology, funding acquisition. **Claudia Pileggi:** visualization, project administration, conceptualization, methodology. **Carlo Tomino:** conceptualization, investigation, formal analysis. **Vincenzo Mollace:** software, funding acquisition, validation. **Sara Ilari:** writing – original draft, methodology, investigation, visualization, project administration. **Carolina Muscoli:** investigation, writing – original draft, project administration, validation.

## Funding

This work was supported by PRIN 2022 cod. 202273HF83. This study is also supported by the Italian Ministry of Health (Ricerca Corrente). Valentina Malafoglia was supported by funding from the Italian Ministry of Health (grants GR‐2021‐12375174), Lucia Carmela Passacatini was supported by funding from the Italian Ministry of Health (grants SG‐2021‐12375551). Open access funding provided by Università degli Studi Magna Graecia di Catanzaro within the CRUI‐CARE Agreement.

## Ethics Statement

The authors confirm that this study is a systematic literature review and did not involve direct participation of patients; therefore, the role of a Principal Investigator with direct clinical responsibility is not applicable.

## Conflicts of Interest

The authors declare no conflicts of interest.

## Data Availability

All data generated/analyzed throughout this research are included in this article.

## References

[1] S. N. Raja , D. B. Carr , M. Cohen , et al., “The Revised International Association for the Study of Pain Definition of Pain: Concepts, Challenges, and Compromises,” Pain 161, no. 9 (2020): 1976–1982, 10.1097/j.pain.0000000000001939. PMC7680716 32694387

[2] A. C. Jesus Palma , C. R. Antunes Júnior , E. S. R. Barreto , et al., “Pharmacological Treatment of Chemotherapy‐Induced Neuropathy: A Systematic Review of Randomized Clinical Trials,” Pain Management Nursing 26, no. 3 (2025): 249–263, 10.1016/j.pmn.2025.01.007. 39952863

[3] S. Ilari , S. Nucera , L. C. Passacatini , et al., “Exploring the Role of Bergamot Polyphenols in Alleviating Morphine‐Induced Hyperalgesia and Tolerance Through Modulation of Mitochondrial SIRT3,” Nutrients 16, no. 16 (2024): 2620, 10.3390/nu16162620. PMC11357234 39203757

[4] S. Ilari , S. Nucera , L. C. Passacatini , et al., “SIRT1: A Likely Key for Future Therapeutic Strategies for Pain Management,” Pharmacological Research 213 (2025): 107670, 10.1016/j.phrs.2025.107670. 39983332

[5] H. L. Edwards , M. R. Mulvey , and M. I. Bennett , “Cancer‐Related Neuropathic Pain,” Cancers 11, no. 3 (2019): 373, 10.3390/cancers11030373. PMC6468770 30884837

[6] S. Ilari , F. Lauro , L. A. Giancotti , et al., “The Protective Effect of Bergamot Polyphenolic Fraction (BPF) on Chemotherapy‐Induced Neuropathic Pain,” Pharmaceuticals 14, no. 10 (2021): 975, 10.3390/ph14100975. PMC8540578 34681199

[7] C. Cioroiu and L. H. Weimer , “Update on Chemotherapy‐Induced Peripheral Neuropathy,” Current Neurology and Neuroscience Reports 17, no. 6 (2017): 47, 10.1007/s11910-017-0757-7. 28421360

[8] C. L. Loprinzi , C. Lacchetti , J. Bleeker , et al., “Prevention and Management of Chemotherapy‐Induced Peripheral Neuropathy in Survivors of Adult Cancers: ASCO Guideline Update,” Journal of Clinical Oncology 38, no. 28 (2020): 3325–3348, 10.1200/JCO.20.01399. 32663120

[9] L. A. Colvin , “Chemotherapy‐Induced Peripheral Neuropathy: Where Are We Now?,” Pain 160, no. Suppl 1 (2019): S1–S10, 10.1097/j.pain.0000000000001540. PMC6499732 31008843

[10] F. Zipp , S. Bittner , and D. P. Schafer , “Cytokines as Emerging Regulators of Central Nervous System Synapses,” Immunity 56, no. 5 (2023): 914–925, 10.1016/j.immuni.2023.04.011. PMC10233069 37163992

[11] V. Malafoglia , S. Ilari , C. Gioia , et al., “An Observational Study on Chronic Pain Biomarkers in Fibromyalgia and Osteoarthritis Patients: Which Role for Mu Opioid Receptor's Expression on NK Cells?,” Biomedicine 11, no. 3 (2023): 931, 10.3390/biomedicines11030931. PMC10046119 36979910

[12] B. Jordan , A. Margulies , F. Cardoso , et al., “Systemic Anticancer Therapy‐Induced Peripheral and Central Neurotoxicity: ESMO‐EONS‐EANO Clinical Practice Guidelines for Diagnosis, Prevention, Treatment and Follow‐Up,” Annals of Oncology 31, no. 10 (2020): 1306–1319, 10.1016/j.annonc.2020.07.003. 32739407

[13] E. M. Smith , H. Pang , C. Cirrincione , et al., “Effect of Duloxetine on Pain, Function, and Quality of Life Among Patients With Chemotherapy‐Induced Painful Peripheral Neuropathy: A Randomized Clinical Trial,” Journal of the American Medical Association 309, no. 13 (2013): 1359–1367, 10.1001/jama.2013.2813. PMC3912515 23549581

[14] Y. Hirayama , K. Ishitani , Y. Sato , et al., “Effect of Duloxetine in Japanese Patients With Chemotherapy‐Induced Peripheral Neuropathy: A Pilot Randomized Trial,” International Journal of Clinical Oncology 20, no. 5 (2015): 866–871, 10.1007/s10147-015-0810-y. 25762165

[15] G. F. Saraceno , D. M. Abrego‐Guandique , R. Cannataro , M. C. Caroleo , and E. Cione , “Machine Learning Approach to Identify Case–Control Studies on ApoE Gene Mutations Linked to Alzheimer's Disease in Italy,” BioMedInformatics 4, no. 1 (2024): 600–622, 10.3390/biomedinformatics4010033.

[16] D. M. Abrego‐Guandique , S. Ilari , S. Nucera , et al., “Vitamin D in the Transition From Acute to Chronic Pain: A Systematic Review,” Nutrients 17, no. 11 (2025): 1912, 10.3390/nu17111912. PMC12156958 40507180

[17] S. Ilari , S. Nucera , V. Malafoglia , et al., “Does Vitamin D Supplementation Impact Fibromyalgia‐Related Pain? A Systematic Review and Meta‐Analysis,” Nutrients 17, no. 20 (2025): 3232, 10.3390/nu17203232. PMC12567182 41156485

[18] R. H. Dworkin , D. C. Turk , K. W. Wyrwich , et al., “Interpreting the Clinical Importance of Treatment Outcomes in Chronic Pain Clinical Trials: IMMPACT Recommendations,” Journal of Pain 9, no. 2 (2008): 105–121, 10.1016/j.jpain.2007.09.005. 18055266

[19] M. Aghili , M. Taherioun , F. Jafari , et al., “Duloxetine to Prevent Neuropathy in Breast Cancer Patients Under Paclitaxel Chemotherapy (a Double‐Blind Randomized Trial),” Supportive Care in Cancer 32, no. 8 (2024): 493, 10.1007/s00520-024-08669-y. 38976095

[20] S. Rokhsareh , S. Haghighi , and M. Tavakoli‐Ardakani , “Evaluating the Effects of Duloxetine on Prophylaxis of Oxaliplatin‐Induced Peripheral Neuropathy in Patients With Gastrointestinal Cancer: A Randomized Double‐Blind Placebo Controlled Clinical Trial,” Journal of Oncology Pharmacy Practice 29, no. 1 (2023): 60–65, 10.1177/10781552211052646. 34738855

[21] H. Matsuoka , S. Iwase , T. Miyaji , et al., “Additive Duloxetine for Cancer‐Related Neuropathic Pain Nonresponsive or Intolerant to Opioid‐Pregabalin Therapy: A Randomized Controlled Trial (JORTC‐PAL08),” Journal of Pain and Symptom Management 58, no. 4 (2019): 645–653, 10.1016/j.jpainsymman.2019.06.020. 31254640

[22] U. Coffeen , M. A. Sotomayor‐Sobrino , A. Jiménez‐González , et al., “Chemotherapy‐Induced Neuropathic Pain Characteristics in Mexico's National Cancer Center Pain Clinic,” Journal of Pain Research 12 (2019): 1331–1339, 10.2147/JPR.S186107. PMC6506570 31118752

[23] S. Y. Song , J. H. Park , J. S. Lee , et al., “A Randomized, Placebo‐Controlled Trial Evaluating Changes in Peripheral Neuropathy and Quality of Life by Using Low‐Frequency Electrostimulation on Breast Cancer Patients Treated With Chemotherapy,” Integrative Cancer Therapies 19 (2020): 1534735420925519, 10.1177/1534735420925519. PMC7273579 32493088

[24] Y. Saito , Y. Takekuma , T. Oshino , and M. Sugawara , “Combination of Mirogabalin and Duloxetine Attenuates Peripheral Neuropathy by Eribulin: A Novel Case Report,” Case Reports in Oncology 15, no. 2 (2022): 606–610, 10.1159/000525059. PMC9247496 35949911

[25] P. Sansone , L. G. Giaccari , C. Aurilio , et al., “Comparative Efficacy of Tapentadol Versus Tapentadol Plus Duloxetine in Patients With Chemotherapy‐Induced Peripheral Neuropathy (CIPN): A Randomized Non‐Inferiority Clinical Trial,” Cancers (Basel) 14, no. 16 (2022): 4002, 10.3390/cancers14164002. PMC9406344 36010995

[26] Y. Singhal , S. K. Pingoliya , A. Mathur , and P. Atal , “A Comparative Study of the Effectiveness and Safety of Pregabalin and Duloxetine in the Treatment of Chemotherapy‐Induced Neuropathic Pain,” Palliative Medicine in Practice 19, no. 2 (2025): 93–100, 10.5603/pmp.100998.

[27] Y. Nakajima , K. Kuribayashi , A. Tada , et al., “Enhancing Duloxetine With Mirogabalin for Treating Taxane‐Induced Peripheral Neuropathy in Advanced Lung Cancer,” Cancer Control 32 (2025): 10732748251353327, 10.1177/10732748251353327. PMC12198551 40556363

[28] A. Kuroyanagi , C. Inano , J. Adachi , G. Kaneko , and H. Toyokawa , “Effective Sympathetic Nerve Block for Chemotherapy‐Induced Peripheral Neuropathy: A Case Report,” Oxford Medical Case Reports 2024, no. 2 (2024): omae006, 10.1093/omcr/omae006. PMC10873712 38370507

[29] J. Rossignol , B. Cozzi , F. Liebaert , et al., “High Concentration of Topical Amitriptyline for Treating Chemotherapy‐Induced Neuropathies,” Supportive Care in Cancer 27, no. 8 (2019): 3053–3059, 10.1007/s00520-018-4618-y. 30607681

[30] C. Zimmermann , N. Swami , M. Krzyzanowska , et al., “Perceptions of Palliative Care Among Patients With Advanced Cancer and Their Caregivers,” CMAJ 188, no. 10 (2016): E217–E227, 10.1503/cmaj.151171. PMC4938707 27091801

[31] H. Obata , “Analgesic Mechanisms of Antidepressants for Neuropathic Pain,” International Journal of Molecular Sciences 18, no. 11 (2017): 2483, 10.3390/ijms18112483. PMC5713449 29160850

[32] F. Porreca , Q. Jiang , and R. J. Tallarida , “Modulation of Morphine Antinociception by Peripheral [Leu5]Enkephalin: A Synergistic Interaction,” European Journal of Pharmacology 179, no. 3 (1990): 463–468, 10.1016/0014-2999(90)90190-H. 2364995

[33] S. Alboghobeish , B. Naghizadeh , A. Kheirollah , B. Ghorbanzadeh , and M. T. Mansouri , “Fluoxetine Increases Analgesic Effects of Morphine, Prevents Development of Morphine Tolerance and Dependence Through the Modulation of L‐Type Calcium Channels Expression in Mice,” Behavioural Brain Research 361 (2019): 86–94, 10.1016/j.bbr.2018.12.020. 30550947

[34] A. Lebel , I. Stambouli , and Y. Boucher , “Effectiveness and Tolerability of Topical Amitriptyline 10% Plus Lidocaine 2% Gel in Adults With Post‐Traumatic Trigeminal Neuropathic Pain: A Real‐World Evidence Study,” Journal of Oral Rehabilitation 53, no. 8 (2026): 1–12, 10.1111/joor.70209. PMC13358445 42087465

[35] M. P. Lunn , R. A. Hughes , and P. J. Wiffen , “Duloxetine for Treating Painful Neuropathy, Chronic Pain or Fibromyalgia,” Cochrane Database of Systematic Reviews 204, no. 1 (2014): CD007115, 10.1002/14651858.CD007115.pub3. PMC10711341 24385423

[36] R. A. Moore , S. Derry , D. Aldington , P. Cole , and P. J. Wiffen , “Amitriptyline for Neuropathic Pain in Adults,” Cochrane Database of Systematic Reviews 7 (2015): CD008242, 10.1002/14651858.CD008242.pub3. PMC6447238 26146793

[37] R. Chow , M. Novosel , O. W. So , et al., “Duloxetine for Prevention and Treatment of Chemotherapy‐Induced Peripheral Neuropathy (CIPN): Systematic Review and Meta‐Analysis,” BMJ Supportive & Palliative Care 13, no. 1 (2023): 27–34, 10.1136/spcare-2022-003815. 36194493

[38] J. P. Reinert , M. A. Veronin , and C. Medina , “Tricyclic Antidepressants in Nociceptive and Neuropathic Pain: A Review of Their Analgesic Properties in Combination With Opioids,” Journal of Pharmacy Technology 39, no. 1 (2023): 35–40, 10.1177/87551225221139699. PMC9899962 36755751

[39] M. J. Ormseth , B. A. Scholz , and C. S. Boomershine , “Duloxetine in the Management of Diabetic Peripheral Neuropathic Pain,” Patient Preference and Adherence 5 (2011): 343–356, 10.2147/PPA.S16358. PMC3150163 21845034

[40] H. C. Gallagher , R. M. Gallagher , M. Butler , D. J. Buggy , and M. C. Henman , “Venlafaxine for Neuropathic Pain in Adults,” Cochrane Database of Systematic Reviews 2015, no. 8 (2015): CD011091, 10.1002/14651858.CD011091.pub2. PMC6481532 26298465

